# Case of Apoplexy in an Infant of Twenty-Two Months

**Published:** 1841-02-27

**Authors:** Francis West


### MEDICAL EXAMINER.

DEVOTED TO MEDICINE, SURGERY, AND THE COLLATERAL SCIENCES.

No. 9.] PHILADELPHIA, SATURDAY, FEBRUARY 27, 1841. [Vol. IV.

### ORIGINAL COMMUNICATION.

*Case of Apoplexy in an Infant of twenty-two months.* By Francis West, M. D.

*Feb.* 18*th*, 1841.—B. R. S., born of healthy parents, æt. twenty-two months; eyes dark; skin very white, and of a waxy appearance; head of usual size and shape; about a year since had a severe catarrh, complicated with some affection of the brain, which, according to the representation of his parents, seriously threatened his life. From that time he continued delicate, and at intervals has suffered from catarrh. In June last he first came under my care for apthous sore-mouth, which continued simple, but severe, for three weeks or more. Soon after recovering from this, symptoms of mild infantile remittent fever set in; he had constant diarrhœa for several days; tumid belly; irregular appetite; cough; great fretfulness, with increase of fever at night; his cough at times was very troublesome, occurring in paroxysms, especially towards evening, which were somewhat relieved by assafœtida and laxatives. During all this period he was dressed and on his mother's lap, his symptoms not being severe enough to confine him to his bed. Under the impression that he might be troubled with worms, he was directed, along with other treatment, gr. vj. of calomel, to be followed by spigelia tea; although no worms were expelled, he seemed to grow better for a time, but the amelioration was only transient. Dentition was regularly and easily accomplished. His temper, naturally irritable, has been more than usually fretful and petulant during the last two or three months. Since he died I have been told by his parents that he occasionally complained of pain in his head, and at times would take his mother's hand and press it upon the part which seemed particularly to trouble him. When free from these fits of pain, which were always short and transient, very little seemed to ail him; and as I was not privy to them, I prescribed nothing in addition to the treatment already mentioned, but the use of syr. sarsap. comp., which was given by me as an alterative, to correct any chronic irritation or alteration, which might possibly be going on, though unrevealed by any decisive symptoms, either in the membranes of the brain or elsewhere. During the twenty four hours preceding the fatal attack of apoplexy, he was observed by his parents to be more than usually lively and well, and seemed better than he had been for six months previously. In the evening, however, of this day, he exhibited more than ordinary fretfulness and temper, for which his father corrected him; he slept quietly, however, through the night. At 8 o'clock on the next morning he called from the chamber to his mother, who was in the room below, and with so natural a voice, that his father detained her from going to him, that he might hear him call again. Very soon after she went up to him she observed a convulsive twitching in the right side of his face, and in a few minutes more the convulsion extended to the arm and leg of the same side. When I saw him, in about half an hour after he was taken, he was perfectly conscious, and followed with his eyes his different friends as they passed through the room: he also took hold of my watch with his left hand, and placed it to his ear. At this time his arm and leg of the side affected with convulsions were found to be perfectly paralyzed; his face was pale; head very hot; pulse rather active, but of natural frequency; pupils obedient to the action of light; no strabismus; skin natural; slight vomiting. Directed him to be leeched to the mastoid regions; cold water to be applied in a stream to his head; warm bath; calomel gr. vj., which he readily swallowed; salt and water injections, and a blister to the back of his neck. The leeching subdued temporarily the twitchings of the face, but had no further influence over the disease. At dinner time, when I saw him again, the convulsions had returned in the right arm and leg, but no twitching of the face; the paralysis still continued; his face was slightly drawn to the left side; consciousness still remained; pupils were natural; blister had drawn; bowels opened several times; no amelioration. Directed ℥iv. of blood by leeches from the temples.

5 *o'clock, P. M.*—Not nearly so conscious, if at all; pupils dilating; both eye-balls quite insensible to the contact of the finger; face perfectly quiet, though there was a sensible deviation of it to the left side; arm and leg as before; his breathing, which hitherto had been natural, now became singultous and sighing; face still pale; skin moist, and of uniform temperature; blisters to insides of legs; has taken calomel gr. ¼ every hour; barley water for drink, which he swallows easily.

11 *o'clock, P. M.*—Symptoms all increased; pupils largely dilated and immoveable; no strabismus. Directed his head to be shaved and blistered; the other blisters have drawn well. No change through the night. Died at 8 o'clock in the morning, just twenty-four hours after the apoplectic seizure.

*Autopsy*, twenty-six hours after death. Present Drs. Pepper and Wallace, and Messrs. Pepper and West. On raising the scalp, a close adhesion by large distended veins about half an inch in extent, was found between it and the cranium at the junction of the sagittal and lambdoidal sutures: the posterior fontanelle was closed; the anterior was open for one-fourth of an inch; except at this point, the sutures were generally closed. On removing the cranium, the arachnoid membrane at the summit of the right hemisphere was minutely injected and traversed by large veins, perfectly filled with dark coagulated blood, which extended over the left hemisphere to about an inch beyond the superior longitudinal sinus. In the course of these vessels the arachnoid was thickened, opaque, and of a yellowish colour. Beneath this membrane, at the summit of the right hemisphere, was a large clot of dark blood, in quantity about ℥j., which dipped down deeply between the convolutions of the brain. In the substance of the right hemisphere above the corpus callosum, several clots of blood were found, the largest of which was about the size of a filbert; the cerebral substance around these coagula, to the extent of some lines, was livid and much softened—the rest of the brain was of the usual consistence in infants; the pink colour of the cortical substance was more marked than common even at this early period of life, and the medullary portion throughout was much dotted with points of blood: there was no softening of the ventricles, nor effusion into their cavities; the plexus choroides was much injected, and the lateral sinuses were distended with very dark blood. There was no thickening of the arachnoid at the base of the brain, nor were any tuberculous deposits found in it at any point. The cerebellum was injected, but without any other alteration. The examination was not extended to any other part of the body.

*Remarks.*—The above case is thus briefly recorded, simply with the view of adding it to the few examples which we already have of extravasation of blood into the cerebral substance of children at so early a period of life. Andral, in his work on "Internal Pathology," when on the subject of the ages at which apoplexy respectively occurs, mentions only three cases of this disease in children under two years, reported by as many different physicians, and we are hence warranted in the conclusion, that by this eminent pathologist its occurrence at this early age was regarded as very rare indeed. He does not mention the particulars of any of the above cases, and we therefore do not know in what they resemble or differ from the one just detailed. Although in this case the effusion of blood into the brain was attended by injection, and some alteration of the membranes, and may therefore be considered to lack the characteristics requisite to constitute it an example of pure, simple, primary apoplexy, it nevertheless, we think, just as conclusively attests the fact of sanguineous effusion into the brain of very young children, as if it had occurred without the complications alluded to.

The effusion, we may presume from the symptoms, &c., was here at the last just as sudden as when it takes place under the more ordinary circumstances of apoplectic seizure: for the softening of the brain which existed, and to which, perhaps, some may be inclined to ascribe it, was as likely to be the effect as the cause of the coagula within its substance; admitting, too, the agency of the meningeal affection in producing them, as it probably did the one on the surface of the brain, the actual result is not the less certain and marked, and it is this after all which forms the peculiar feature of the case, and which has induced its publication. We have dwelt most particularly upon the extravasation of blood into the substance of the brain, because, by some pathologists we believe, such a lesion only is recognised as constituting *proper* apoplexy; and to this restricted sense of the term, as well as to that which includes within its meaning all effusions of blood interesting the brain, the facts of the case presented equally well apply. It is now matter of regret that the other cavities of the body were not examined; at the period of the autopsy, no such disposition of the case as the present was anticipated, and therefore the account of it is deficient in many points of critical interest. Had evidences of disease, however, in the alimentary canal or elsewhere been found, it could only have been the remote cause of the apoplexy, and therefore the singularity of the lesion of the brain would not have been affected by any such discovery. In conclusion we may remark, that we have thus simply recorded the case, not with any expectation that by itself it will be at all useful, for such a property rarely attaches to individual and insulated cases, but only with the view of contributing it to the general stock of materials upon the same subject, from which, by those capable of the task of analysing and comparing them, we can alone hope to draw any positively useful information in regard to it.

